# Post-cesarean Sepsis Secondary to Vernix Caseosa Peritonitis: A Diagnostic Challenge With Misleading Imaging

**DOI:** 10.7759/cureus.104833

**Published:** 2026-03-07

**Authors:** Nada A Hassane, Maral Azarayesh, Sajed M Nabi, Sami Alghayath, Nada Alsuwaidi, Hind O Al Shafar, Ali Reza

**Affiliations:** 1 Medicine, Mohammed Bin Rashid University of Medicine and Health Sciences, Dubai, ARE; 2 Medicine and Surgery, Mohammed Bin Rashid University of Medicine and Health Sciences, Dubai, ARE; 3 General and Colorectal Surgery, Mediclinic Parkview Hospital, Dubai, ARE

**Keywords:** diagnostic delay, foreign body reaction, post-op complication, postpartum sepsis, vernix caseosa peritonitis

## Abstract

Vernix caseosa peritonitis is an uncommon but important cause of postoperative intra-abdominal sepsis following cesarean delivery, resulting from a sustained foreign body inflammatory reaction to fetal vernix introduced into the maternal peritoneal cavity, typically manifesting within hours to days after surgery and often mimicking other acute surgical abdominal conditions with non-specific or misleading radiological findings. We report the case of a 43-year-old woman who presented five days after an uncomplicated cesarean section with severe abdominal pain, vomiting, and markedly elevated inflammatory markers, in whom initial computed tomography of the abdomen and pelvis suggested enteritis with associated free intraperitoneal fluid, without the characteristic features of vernix caseosa peritonitis. Despite broad-spectrum intravenous antibiotics, her condition deteriorated with the development of progressive pelvic collections requiring surgical intervention, and intraoperative findings revealed multiple yellow patches on the serosal surfaces of visceral organs consistent with vernix caseosa peritonitis, with the diagnosis subsequently confirmed on histopathological examination. This case highlights the significant diagnostic challenge posed by this rare entity and underscores the importance of maintaining a high index of clinical suspicion in patients presenting with peritonitis following cesarean delivery, as timely surgical source control via laparotomy and peritoneal lavage remains the cornerstone of management.

## Introduction

Vernix caseosa peritonitis (VCP) is a rare but clinically important cause of postpartum acute abdomen, most commonly reported following cesarean delivery [1,2]. Vernix caseosa is a physiological substance composed of anucleate fetal squamous epithelial cells (corneocytes) and sebaceous material, serving a protective role for fetal skin in utero and during delivery [3]. The incidence of VCP is extremely low, with only isolated case reports and small case series described in the literature, contributing to limited clinical awareness and frequent diagnostic delay [1,4].

The pathophysiology of VCP is believed to involve spillage of vernix-containing amniotic fluid into the maternal peritoneal cavity during cesarean section, where it elicits a foreign-body granulomatous inflammatory reaction [5]. This inflammatory process may result in diffuse peritonitis, intra-abdominal collections, and a pronounced systemic inflammatory response, often mimicking more common surgical or infectious causes of postpartum acute abdomen, such as appendicitis, bowel perforation, or pelvic abscess [6,7]. Reported risk factors are poorly defined; however, cesarean delivery remains the most consistently identified association, with additional reported links to prolonged rupture of membranes, meconium-stained amniotic fluid, and complicated or traumatic deliveries, although many cases occur following otherwise uncomplicated procedures [5,8]. Owing to its non-specific clinical, laboratory, and radiological features, VCP is frequently recognized only at surgical exploration or on histopathological examination, and delayed diagnosis may result in unnecessary procedures and increased morbidity [4]. We report a case of vernix caseosa peritonitis in a postpartum patient who developed severe abdominal pain and a marked systemic inflammatory response several days following cesarean delivery, highlighting the diagnostic challenges associated with this rare entity and emphasizing the importance of considering VCP in the differential diagnosis of post-cesarean acute abdomen.

## Case presentation

A 43-year-old gravida five para five (G5P5) woman presented to the emergency department five days after an uncomplicated lower-segment cesarean section performed for placenta previa. She had been discharged home the previous day. Since surgery, she had experienced persistent abdominal pain that had significantly worsened over the preceding 24 hours. This was associated with nausea and two episodes of large-volume vomiting, each estimated at approximately 1-1.5 liters. The vomitus was described as dark green-black in color. She denied fever, diarrhea, melena, hematochezia, urinary symptoms, or syncope, but reported reduced oral intake and marked fatigue.

Her medical history was notable for iron-deficiency anemia, with a baseline hemoglobin concentration of approximately 10 g/dL. Her hemoglobin level at discharge following cesarean delivery was 8.8 g/dL. Postoperatively, she had been prescribed regular diclofenac for analgesia and prophylactic enoxaparin.

On examination, she was alert and oriented but appeared pale and unwell. She was tachycardic, with a heart rate of 110-120 beats per minute, while blood pressure and oxygen saturation were preserved. She was afebrile. Abdominal examination revealed diffuse tenderness, most pronounced in the epigastric region and right lower quadrant, with mild abdominal distension. There was no guarding or rigidity, and bowel sounds were present. The cesarean section wound was clean, dry, and intact. Cardiovascular and respiratory examinations were otherwise unremarkable.

Laboratory investigations demonstrated a marked inflammatory response, with a raised white cell count, neutrophilia, and an elevated C-reactive protein (CRP) and procalcitonin (PCT). Renal function, electrolyte levels, and liver enzymes were within normal limits. Venous blood gas analysis showed a normal pH with elevated lactate. These findings are summarized in Table 1.

**Table 1:** Results of baseline laboratory investigations Estimated glomerular filtration rate (eGFR), calculated using the Chronic Kidney Disease Epidemiology Collaboration (CKD-EPI) equation. Abnormal values are highlighted in bold. HPF: high-power field.

Laboratory Test	Result (Admission, Day 1)	Result (Discharge, Day 29)	Reference Range
Hematology			
White blood cell count	16.0	5.9	4.0-11.0 ×10³/µL
Hemoglobin	10.6	9.3	11.5-16.0 g/dL
Hematocrit	34.3	30.5	31%-42%
Mean corpuscular volume	76.2	75	80-100 fL
Platelet count	325	340	150-450 ×10³/µL
Inflammatory markers			
C-reactive protein	415	11.9	0.0-5.0 mg/L
Procalcitonin	Not tested	0.21	<0.05 ng/mL
Urea and electrolytes			
Sodium	144.2	Not repeated	136-145 mmol/L
Potassium	4.08	Not repeated	3.5-5.1 mmol/L
Chloride	106.2	Not repeated	98-107 mmol/L
Bicarbonate	19.0	Not repeated	22-28 mmol/L
Renal function tests			
Creatinine	50.9	Not repeated	53-97 µmol/L
eGFR (CKD-EPI)	115	Not repeated	>60 mL/min/1.73 m²
Liver function tests			
Alanine aminotransferase	15.3	Not repeated	<35 U/L
Aspartate aminotransferase	26.4	Not repeated	<35 U/L
Alkaline phosphatase	142	Not repeated	35-104 U/L
Direct bilirubin	7.59	Not repeated	≤7.0 µmol/L
Urinalysis			
Protein	Positive (3+)	Not repeated	Negative
Ketones	Positive (2+)	Not repeated	Negative
Red blood cells	3-5	Not repeated	0-2/HPF
Leukocytes	6-10	Not repeated	0-5/HPF
Specific gravity	1.050	Not repeated	1.003-1.035

**Figure 1:**
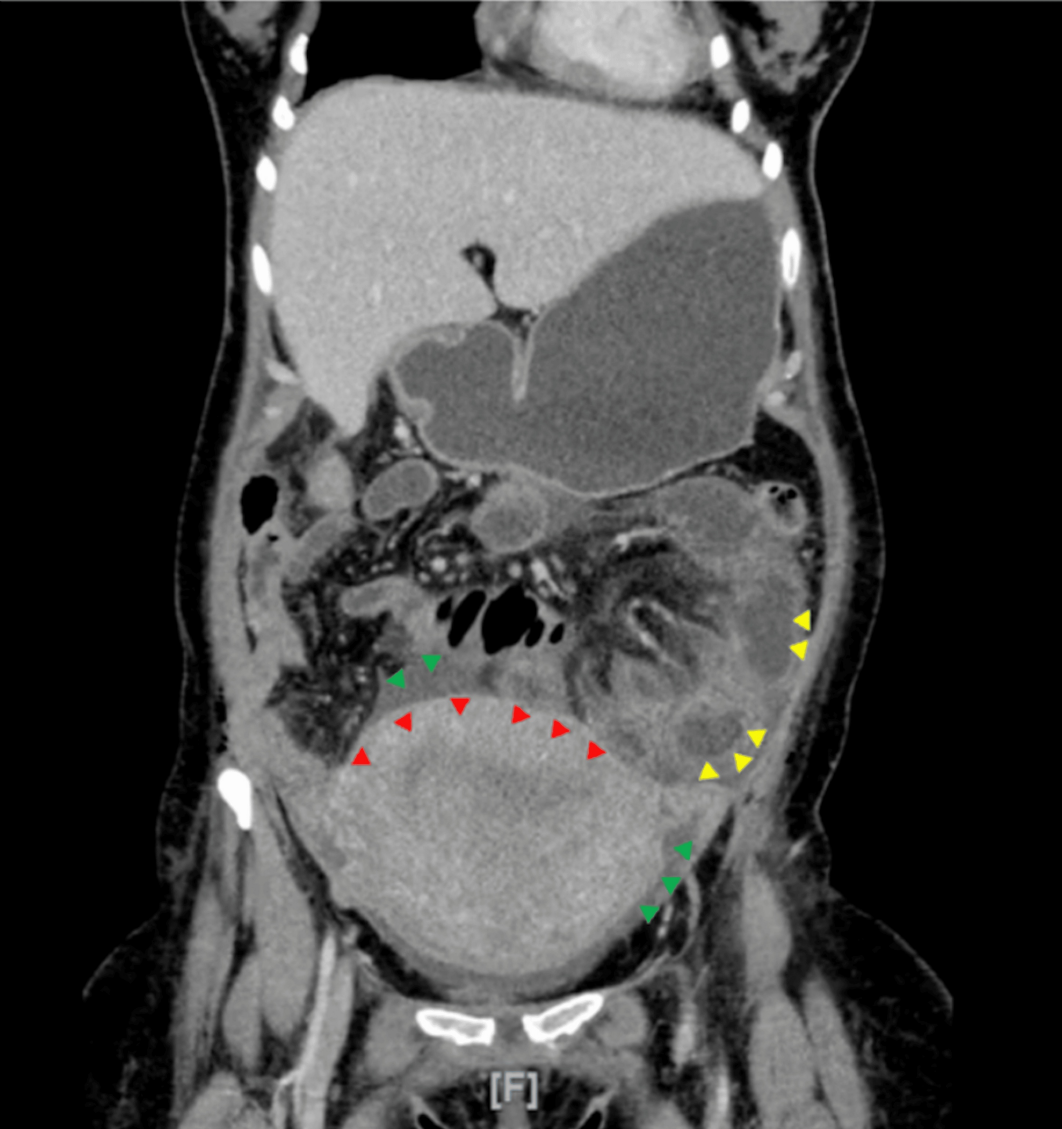
Coronal slice through contrast-enhanced CT abdomen and pelvis Performed on the day of readmission, corresponding to post-cesarean section day 4, showing red arrowheads, enlarged uterus consistent with the postpartum period; green arrowheads, pockets of free fluid in the pelvis; and yellow arrowheads, diffuse marked wall thickening and mild dilatation observed in the small bowel loops, consistent with enteritis.

Radiological investigations initially included point-of-care ultrasound, which demonstrated free fluid in the pelvis. Subsequently, a contrast-enhanced CT scan of the abdomen and pelvis, performed on day 1 of readmission, demonstrated diffuse wall thickening and mild dilatation of the jejunal and proximal ileal loops, consistent with enteritis (Figure 1). The uterus appeared diffusely enlarged in keeping with the postpartum period. A mild-to-moderate volume of free intraperitoneal fluid was present, with no focal collections identified. Minimal bilateral pleural effusions and a trivial pericardial effusion were also noted.

**Figure 2:**
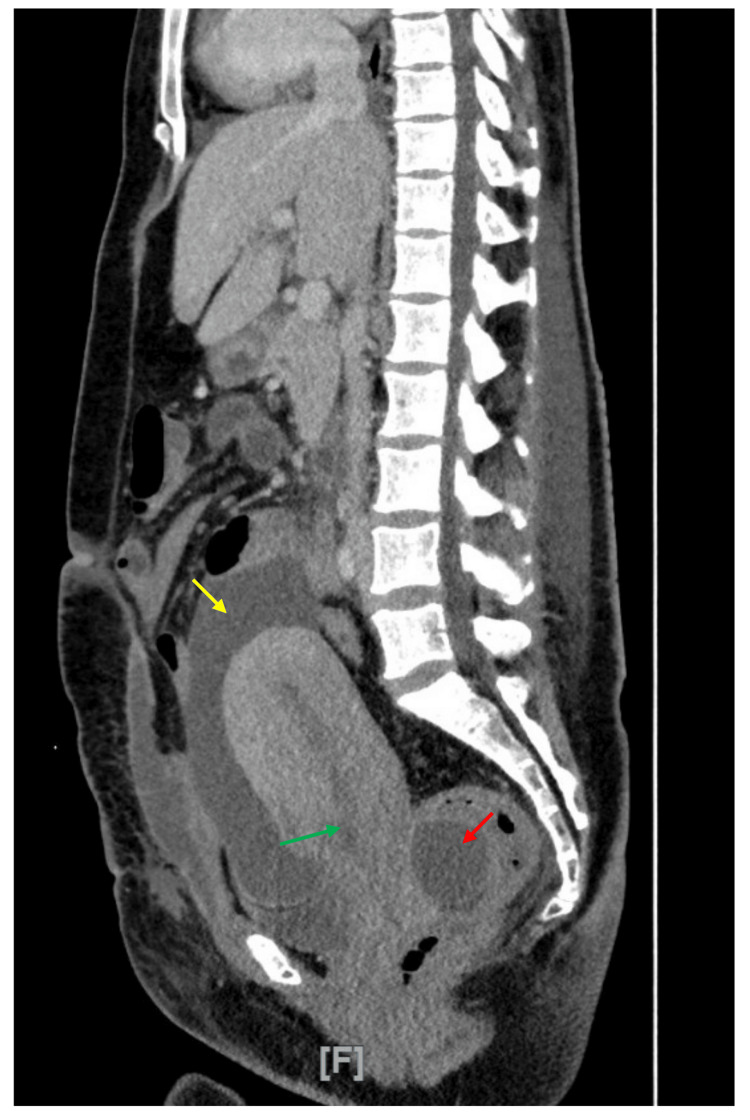
Repeat sagittal contrast-enhanced CT of the abdomen and pelvis on day 6 Yellow arrow indicates localized collection posterior to the cervix with a thick enhancing wall measuring approximately 6.6 x 3.8 cm. Green arrow indicates hypoattenuation defect in the anteroinferior uterine wall consistent with a cesarean section scar. Red arrow indicates larger fluid collection superior and anterior to the uterus.

**Figure 3:**
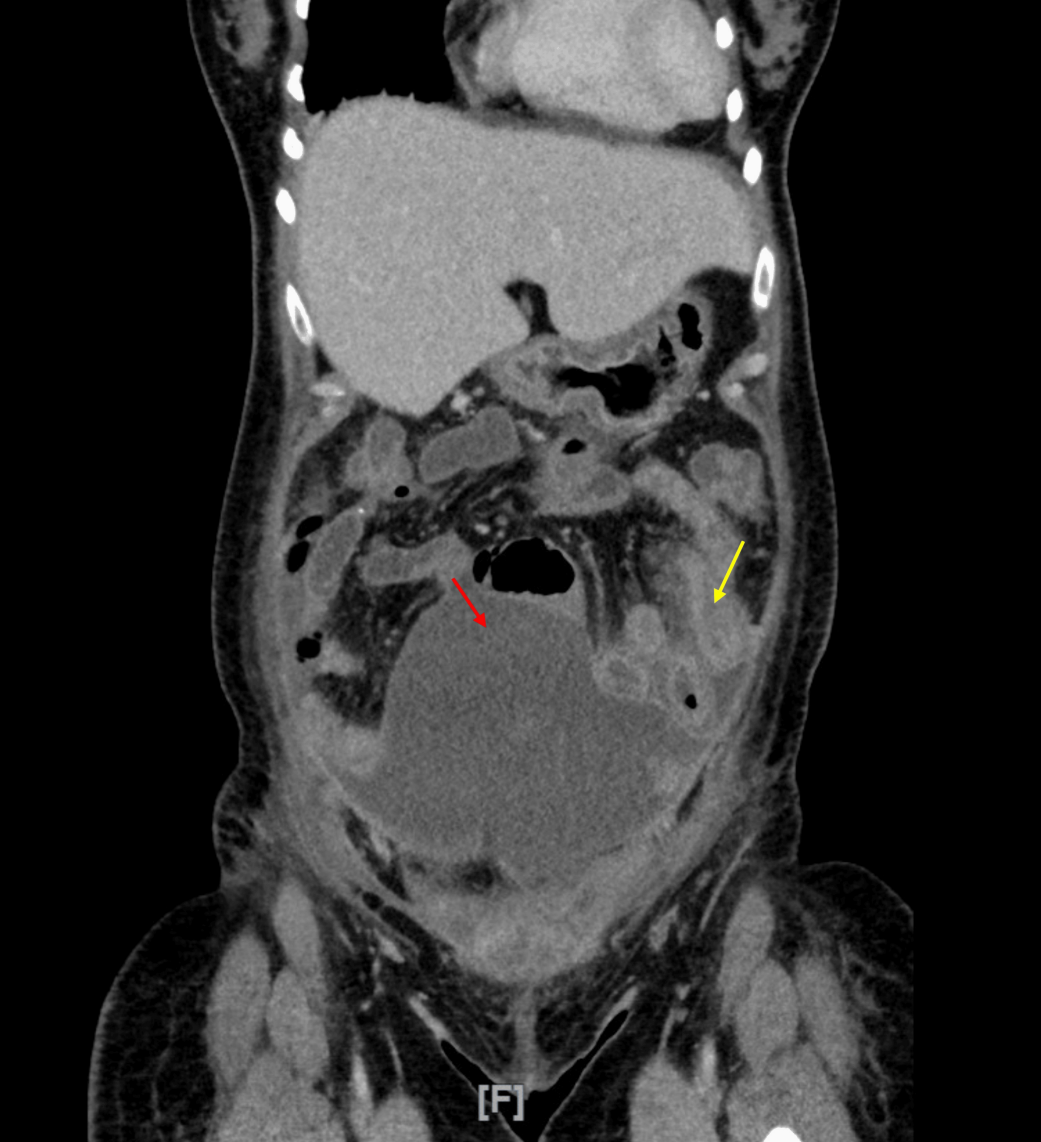
Repeat coronal slice through contrast-enhanced CT abdomen and pelvis from day 6 of admission ⁠Red arrow indicates a large fluid collection superior and anterior to the uterus, measuring 15.8 cm in the craniocaudal dimension and 6 cm in the anteroposterior dimension. The wall of the collection demonstrated contrast enhancement. There was communication with a collection in the parietal wall. Yellow arrow indicates secondary inflammation of the adjacent bowel, demonstrating wall thickening and surrounding fat stranding.

Repeat CT scan of the abdomen and pelvis with intravenous contrast on day 6 demonstrated interval progression, with development of an elongated organized fluid collection surrounding the uterus, predominantly anteriorly and superiorly, measuring approximately 15.8 cm in craniocaudal dimension and 6 cm in anteroposterior dimension, with an enhancing wall (Figures 2, 3). A communicating collection was seen within the anterior abdominal wall at the cesarean section site. A further localized collection was identified posterior to the cervix, measuring approximately 6.6 × 3.8 cm. Mild free fluid was present in the perihepatic and perisplenic regions. Increasing bilateral pleural effusions were also observed.

The differential diagnosis included postoperative ileus, enteritis, appendicitis, intra-abdominal sepsis, pelvic abscess, and bowel injury. In retrospect, vernix caseosa peritonitis was recognized as the underlying diagnosis. The patient was admitted with a working diagnosis of abdominal sepsis and commenced on intravenous fluid resuscitation, proton pump inhibitor infusion, antiemetics, analgesia, and broad-spectrum intravenous antibiotics; the choice was piperacillin-tazobactam. Despite medical treatment, her abdominal pain persisted, and inflammatory markers remained elevated. Clinically, she remained tachycardic and systemically unwell, and radiological progression of pelvic collections was demonstrated on repeat CT imaging.

**Figure 4:**
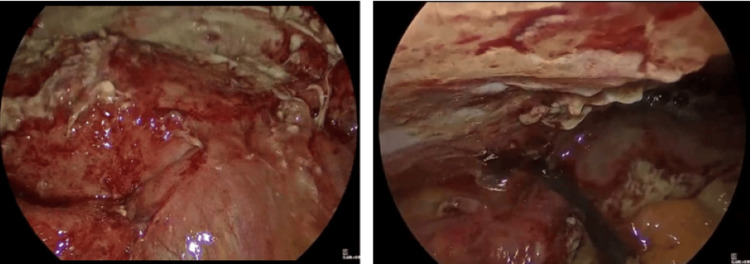
Intraoperative laparoscopic views Showing vernix caseosa peritonitis, characterized by diffuse inflammatory reaction with “cheesy, curd-like” white deposits coating the peritoneal surfaces and visceral organs, mimicking purulent peritonitis.

In view of ongoing sepsis and failure of conservative management, she underwent an exploratory laparotomy. Intraoperatively, purulent inflammatory material consistent with vernix caseosa was identified within the peritoneal cavity, without evidence of bowel perforation. Drainage of intra-abdominal collections was performed, followed by diagnostic laparoscopy (Figure 4), extensive peritoneal lavage, adhesiolysis, and appendicectomy.

Microbiological cultures obtained from blood and peritoneal fluid showed no growth. Cervical polymerase chain reaction (PCR) testing detected *Mycoplasma hominis* and *Ureaplasma parvum*, which were considered contributory but not causative. Following surgical source control, the patient demonstrated gradual clinical and biochemical improvement and was discharged with outpatient follow-up.

## Discussion

This case describes a postpartum patient who presented five days following a cesarean section with severe abdominal pain, persistent vomiting, tachycardia, and a marked systemic inflammatory response. Initial laboratory investigations demonstrated leukocytosis and significantly elevated inflammatory markers, while early computed tomography revealed free intraperitoneal fluid and was interpreted as enteritis, resulting in diagnostic delay. Despite broad-spectrum intravenous antibiotic therapy, the patient deteriorated clinically, and repeat imaging demonstrated evolving intra-abdominal collections. Exploratory laparotomy subsequently confirmed vernix caseosa peritonitis (VCP), and the patient showed significant clinical improvement following extensive peritoneal washout and drainage.

Vernix caseosa peritonitis remains an uncommon but important diagnostic consideration in postpartum patients presenting with acute abdominal symptoms following cesarean delivery [1,2,9-11]. The rarity of the condition and its overlap with more common surgical and infectious causes of postpartum acute abdomen frequently lead to misdiagnosis and delayed definitive management [2,4,7]. In previously reported cases, VCP has most often been initially attributed to appendicitis, bowel injury, pelvic abscess, or generalized intra-abdominal sepsis, reflecting the non-specific nature of its clinical presentation and early investigative findings [2,5,6].

The clinical features observed in this case are consistent with those reported in the literature. Most cases present within days to weeks postpartum, with abdominal pain being the most common symptom, often accompanied by vomiting, tachycardia, leukocytosis, and markedly elevated inflammatory markers [1,5,8]. Fever may be absent or delayed, as observed in our patient, and this has been repeatedly reported as a contributor to diagnostic difficulty and delayed diagnosis [5]. Consequently, initial clinical suspicion is frequently directed toward more common postpartum or surgical conditions [6,7].

Radiological evaluation represents a major diagnostic challenge in VCP. Computed tomography findings are variable and non-specific, commonly demonstrating free intraperitoneal fluid, fat stranding, bowel wall thickening, or loculated collections, which may mimic enteritis or other intra-abdominal pathology [12]. Multiple reports describe initially reassuring or misleading imaging, with diagnosis only becoming apparent following clinical deterioration or repeat imaging [12,13]. This pattern was mirrored in the present case, where early CT findings delayed definitive diagnosis and surgical intervention.

Although several associations have been described, risk factors for vernix caseosa peritonitis remain poorly defined, with cesarean delivery representing the most consistently reported context in the literature [2,5,8]. Importantly, many reported cases, including the present case, occur following otherwise uncomplicated cesarean sections, underscoring that the absence of identifiable obstetric risk factors does not exclude the diagnosis. Moreover, although rare, VCP has been reported after normal vaginal delivery, with retrograde flow through the fallopian tubes considered as a possible mechanism [14,15].

Diagnostic evaluation of suspected VCP typically demonstrates laboratory evidence of significant systemic inflammation, including leukocytosis and markedly elevated inflammatory markers, while microbiological cultures are frequently negative, reflecting the predominantly inflammatory rather than infectious nature of the condition [1,8,10]. Imaging modalities such as ultrasound and computed tomography are commonly employed to evaluate postpartum abdominal pain, but findings are often non-specific, necessitating repeat imaging when clinical deterioration persists [12,13]. Definitive diagnosis is most commonly established intraoperatively, with histopathological examination demonstrating a foreign-body granulomatous reaction containing anucleate fetal squamous cells and keratin debris consistent with vernix caseosa [5,11].

Management of VCP centers on timely surgical intervention once conservative measures fail or diagnostic uncertainty persists. Peritoneal lavage with removal of inflammatory material and drainage of collections has been consistently associated with rapid clinical improvement in reported cases [1,10,11]. Broad-spectrum antibiotics are commonly initiated empirically due to concern for postpartum sepsis, but should be regarded as adjunctive therapy rather than definitive management, as antibiotic therapy alone is often insufficient [8,10]. In the present case, significant clinical improvement occurred only after exploratory laparotomy with extensive peritoneal washout and drainage, consistent with outcomes reported in the literature.

Vernix caseosa peritonitis should always be considered in the differential diagnosis of postpartum abdominal pain and sepsis in patients with recent cesarean delivery, particularly when inflammatory markers are markedly elevated, and imaging findings are inconclusive. Early recognition and prompt surgical management are essential to reducing morbidity and preventing unnecessary extensive surgical procedures.

## Conclusions

Vernix caseosa peritonitis is a rare but clinically important cause of postoperative peritonitis following cesarean delivery, resulting from a sterile foreign-body inflammatory reaction rather than an infection. Owing to its non-specific clinical and radiological features, diagnosis is frequently delayed or initially misattributed to intra-abdominal sepsis. Awareness of this entity and its inclusion in the differential diagnosis are crucial, as prompt recognition and appropriate surgical peritoneal lavage can lead to rapid resolution and help avoid unnecessary prolonged antibiotic therapy or invasive interventions.
